# Pharmacist’s Support in the Transition From Hospital to Home Care in Patients With Multiple Medications: Avoidance of Fragmentation of Care

**DOI:** 10.7759/cureus.87751

**Published:** 2025-07-11

**Authors:** Youtaro Arima, Kazuhiro Sumitomo, Yasuo Miyauchi, Tsuneyuki Takahashi, Katsutoshi Furukawa

**Affiliations:** 1 Department of Pharmacy, Wakabayashi Hospital, Tohoku Medical and Pharmaceutical University, Sendai, JPN; 2 Department of General Medicine, Wakabayashi Hospital, Tohoku Medical and Pharmaceutical University, Sendai, JPN; 3 Department of Respiratory Medicine, Wakabayashi Hospital, Tohoku Medical and Pharmaceutical University, Sendai, JPN; 4 Division of Geriatric and Community Medicine, Tohoku Medical and Pharmaceutical University, Sendai, JPN

**Keywords:** compliance, geriatrics, medication, pharmacist, polypharmacy

## Abstract

Transitions from hospital to home care are high-risk periods, often leading to medication errors and care fragmentation. This case report highlights the essential role of pharmacists in ensuring safe medication transitions and continuity of care, particularly through the use of the simple suspension method (SSM), a technique that simplifies the administration of medication for patients with dysphagia. The case involves a woman in her 80s with multiple chronic conditions and swallowing difficulties, hospitalized for pneumonia. During her hospital stay, a pharmacist introduced and monitored SSM, adjusted her medication regimen, and reduced polypharmacy to ease the caregiving burden. Collaborative planning with nurses ensured a simplified post-lunch medication schedule, and the patient’s daughter, as primary caregiver, was educated in administering SSM. Upon discharge, medication information was shared with the community pharmacy to support continuity. Follow-up by community pharmacists and care staff confirmed successful medication management and SSM use at home. This case underscores the pivotal role of pharmacists in personalized medication management, care coordination, and patient-family support. It also suggests that integrating pharmacist-led interventions with emerging technologies such as Artificial Intelligence and Internet of Things can enhance care for older adults, especially those living alone, and potentially reduce hospital readmissions.

## Introduction

Transitions from hospital to home care are critical and challenging because of the potential medication errors, leading to severe adverse outcomes such as “fragmentation of care” including “hemorrhage due to anticoagulant dosing errors” or “hypoglycemia from antidiabetic medication errors” [1,2] in patients. This necessitates the need for a safe and steady transfer of medication-related information during these transitions [3]. Collaborative care by general practitioners and pharmacists is important and effective in preventing fragmentation of care [4]. Additionally, strengthened collaboration between hospital staff and community pharmacists is essential for improving medication management and patient care, emphasizing the importance of information sharing [5]. The simple suspension method (SSM), involving tablet or capsule administration via a tube after disintegration and suspension in hot water without crushing or opening the capsule, has recently been utilized for patients with dysphagia, particularly those with cancer [6]. In the SSM method we employed, drugs are delivered after their natural dissolution and suspension by soaking them in warm water at 55℃ for 10 minutes, without any processes of crushing or removal of capsules. This report highlights the critical role of pharmacists in medication management, prevention of care fragmentation, and support during transitions to home care. It emphasizes the effectiveness of pharmacist-led interventions and the expanding role of pharmacists in home-based care. Additionally, we highlighted the pivotal role of pharmacists in facilitating a safe and effective transition to home care, focusing particularly on managing and reducing polypharmacy using the SSM.

## Case presentation

On April 8, 2024, an older woman in her 80s with a medical history of hypertension, rheumatoid arthritis, diabetes, and swallowing difficulty, requiring the administration of all oral medications in a crushed form, was admitted to our hospital due to pneumonia. Her dysphagia actually had been present prior to hospitalization and the difficulty in swallowing was getting worse during the admission. Upon admission, the inpatient ward pharmacist evaluated the patient's medication regimen and introduced the SSM to simplify medication management. Furthermore, the pharmacist continuously assessed the appropriateness of the SSM during her hospital stay, making necessary adjustments to medications. The standard SSM approach, in which a thickening agent is used to assist swallowing, was implemented throughout the hospitalization. Her pneumonia improved following treatment with ceftriaxone (1 g every 12 h) and micafungin (75 mg every 24 h). Actually, her body temperature, white blood cell count, and C-reactive protein changed from 38.8℃ to 36.7℃, 15,400 to 6,230 /μL and 8.53 to 0.54 mg/dL, respectively. Subsequently, a plan was developed for transition from a hospital to home-based care supported by short-term admission to a nursing home. Although predicting her long-term prognosis was challenging during care planning, personalized medication management was discussed based on her health status and support needs. The attending doctor and the pharmacist decided to continue only essential medications to prevent polypharmacy and reduce patient and caregiver burden. Additionally, the pharmacist worked closely with the inpatient ward nurses to consolidate her medication schedule, ensuring that all oral medications (except those before bedtime) should be administered after lunch. This approach also aimed to simplify medication management for her daughter, the primary caregiver of the patient, who expressed relief and reassurance. The daughter received comprehensive instructions on the SSM, which enhanced her confidence in continuing the regimen at home. Although direct requests or feedback from the patient was unavailable due to her communication difficulties caused by dementia, family support played a crucial role in her transition and medication management. Of the 12 medications, which the patient took orally (Table 1), prescribed at the time of admission, the regimen was reduced to seven by discharge, substantially alleviating the burden on both the patient and her family. Indeed, the patient’s daughter reported that the reduction in the number of medications led to a reduction in the care burden. Upon discharge, relevant information was provided to the patient’s insurance pharmacy, along with a request to monitor the continued use of the SSM. A post-discharge care conference with hospital staff, community pharmacists, and nursing home personnel confirmed that the patient's medications were being properly managed. Continued communication between the community pharmacy and the physicians indicated a further understanding of the patient’s condition between each other, no changes to her prescriptions, and that the pharmacist actively monitored the implementation of the SSM after discharge. Although this pharmacist-led follow-up was crucial in ensuring continuity of care, the presence of care staff facilitated the easier implementation of this approach.

**Table 1:** The list of medications upon admission and at discharge. The number of medications were decreased from 12 to seven.

	(A) Medication upon admission	Indication		(B) Prescription at discharge	Indication
1	Dimemorfan phosphate 10 mg, 1 tablet, 3 times a day, after meal	Cough	1	Iron polysaccharide syrup, 10 mL, once a day, after lunch	Iron-deficiency anemia
2	Tranexamic acid 250 mg, 1 tablet, 3 times, once a day, after each meal	Tonsillitis, Laryngitis	2	Ramelteon 8 mg, 1 tablet, once a day, before bedtime	Insomnia
3	Telmisartan 20 mg, 1 tablet, once a day, after breakfast	Hypertension	3	Prednisolone 5mg, 1 tablet, once a day, after lunch	Rheumatoid arthritis
4	Amlodipine orally disintegrating 5 mg, 1 tablet, once a day, after breakfast	Hypertension	4	Magnesium oxide 250 mg, 2 tablets, once a day, after lunch	Constipation
5	Nitrazepam granules 1%, 0.5 g, once a day, before bedtime	Insomnia	5	Furosemide 20 mg, 1 tablet, once a day, after lunch	Hypertension
6	Prednisolone 5 mg, 1 tablet, once a day, after breakfast	Rheumatoid arthritis	6	Insulin degludec 20 units, once a day, before bedtime	Diabetes
7	Magnesium oxide granules 0.33 g, 3 times a day, after each meal	Constipation	7	Elneopa NF No.1 injection, once a day	Dehydration, Malnutrition
8	Gliclazide 20 mg, 1 tablet, once a day, after breakfast	Diabetes			
9	Imeglimin 500 mg, 1 tablet, once a day, after breakfast	Diabetes			
10	Atorvastatin 10 mg, 1 tablet, once a day, after breakfast	Hypercholesterolemia			
11	Carbocisteine dry syrup 50%, 0.5 g, twice a day, after breakfast and dinner	Tonsillitis, Laryngitis			
12	Precipitated calcium carbonate/cholecalciferol/magnesium carbonate, 2 tablets, once a day, after breakfast	Hypocalcemia			

## Discussion

This case demonstrates the pivotal role of pharmacists in easing the transition from hospital admission to home care and reducing the strain on the patient and her family by decreasing the number of medicines, utilizing the SSM. Implementing SSM in close collaboration with community pharmacists helped maintain continuity of care. Community pharmacists and care staff play a crucial role in medication management for older adults who live alone. Furthermore, addressing these challenges through emerging technologies, such as Artificial Intelligence (AI)-powered telemedicine platforms [7] and Internet of Things (IoT) devices [8], could offer promising solutions especially for patients living alone. Importantly, follow-up care by visiting pharmacists can reduce hospital readmission rates, suggesting that a combination of technology and human support can further improve care management of older patients [9]. This case report emphasizes the crucial role of pharmacists in tailor-made medication, care organization, and patient-family relationships. We-organized contributions by pharmacists with new-age technologies such as AI and IoT are able to improve medications and care for older adults and their quality of life.

## Conclusions

This case report highlights a pharmacist's crucial role in a safe hospital-to-home transition for an elderly woman with multiple conditions including dysphagia. The pharmacist implemented SSM, adjusted her medications to reduce polypharmacy, and collaborated with nurses for a simplified schedule. The patient's daughter received SSM training, and the community pharmacy was informed for continuity. Follow-up confirmed successful medication management, underscoring the value of pharmacist-led interventions and potential of technology in enhancing care. In conclusion, this case underscores the vital role of pharmacists in ensuring safe care transitions through medication optimization, caregiver education, and effective interprofessional collaboration, demonstrating how pharmacist-led interventions and supportive technology can enhance continuity and quality of care.
